# CDC To Release Updated Emerging Infectious Disease Plan

**DOI:** 10.3201/eid0403.980354

**Published:** 1998


### CDC To Release Updated Emerging Infectious Disease Plan

Preventing Emerging Infectious Diseases: A Strategy for the 21st Century outlines new measures toward achieving emerging infectious disease prevention and control. The updated plan signals the second phase of the campaign launched in 1994 with the publication of Addressing Emerging Infectious Disease Threats: A Prevention Strategy for the United States, a collaborative effort of the Centers for Disease Control and Prevention under the leadership of the National Center for Infectious Diseases and institutions and agencies throughout the United States and abroad.

The objectives and activities in the updated plan are organized under the same four goals described in the 1994 publication: surveillance and response, applied research, infrastructure and training, and prevention and control. Nine specific priority program areas are outlined: antimicrobial resistance; foodborne and waterborne diseases; vector-borne and zoonotic diseases; diseases transmitted through blood transfusions or blood products; chronic diseases caused by infectious agents; vaccine development and use; diseases of people with impaired host defenses; diseases of pregnant women and newborns; and diseases of travelers, immigrants, and refugees.

Achieving the goals outlined in the updated plan will continue to require sustained and coordinated efforts of agencies and organizations, state and local health departments (surveillance of infectious diseases), academic centers and other federal agencies (research), health-care providers and health-care networks (guideline development and dissemination), international organizations (outbreak responses overseas), and other partners.

The executive summary of Preventing Emerging Infectious Diseases: A Strategy for the 21st Century will be released as a special issue of the Morbidity and Mortality Weekly Report on September 10, 1998. An electronic version of the full document as well as information on how to order a print copy will be available shortly afterwards at http://www.cdc.gov/ncidod/ncid.htm.

### First Congress of the European Society for Emerging Infections, September 13-16, 1998, Budapest, Hungary

Founded in 1997 by human and veterinary infectious disease specialists, the European Society for Emerging Infections (ESEI) forms a European network for the study of new or emerging infectious diseases. This interdisciplinary forum was a necessity because most emerging infections are zoonoses or are linked with animal care or with animal product handling. ESEI is holding its first International Congress in the Atrium Hyatt Conference Centre, Budapest, Hungary, September 13–16, 1998. The opening lecture, "Emerging infections—an overview," will be given by Prof. Luc Montagnier. The meeting will consist of invited lectures, two free paper sessions, a roundtable discussion, and daily poster presentations. Conference topics include risk factors for emergence of pathogens, tick-borne diseases, hantavirus infections, transmissible spongiform encephalopathies, Borna disease, lyssavirus infections, and foodborne diseases. A banquet cruise on the Danube will end the Congress on Wednesday evening, September 16, 1998.

Abstracts should address one of the above topics and be submitted before the deadline of May 31, 1998. For more information, please contact ESEI President Prof. M. Granström, Microbiology, Karolinska Hospital, S-171 76 Stockholm, Sweden; fax: 46-8-30-80-99; e-mail: marta@mb.ks.se or the local organizer Dr. A. Lakos, Centre for Tick-borne Diseases, Visegradi 14, H-1132 Budapest, Hungary, fax: 36-1-349-49-26, e-mail: alakos@helka.iif.hu.

### Foodborne Illness: A Disease for All Seasons, October 27 and 28, 1998, Newark, Delaware

Sponsored by the Public Health Laboratories of Delaware, Maryland, New Jersey, and Pennsylvania and the National Laboratory Training Network, Eastern Office, this seminar will provide up-to-date information on changes in epidemiology in foodborne diseases, emerging infectious organisms, proper food and clinical specimen collection and testing, and strategies to decrease foodborne illness. Speakers will represent the Centers for Disease Control and Prevention, the Food and Drug Administration, Minnesota Department of Agriculture, Minnesota Department of Public Health, and the University of Maryland.

For more information, contact Christine Ford, National Laboratory Training Network, Eastern Office, Delaware Public Health Laboratory; tel.: 302-653-2841; fax: 302-653-2844; e-mail: ford115w@cdc.gov.

### December 1998 International Conference on Antiretroviral Therapy, St. Thomas, West Indies

The International Medical Press will sponsor the International Conference on the Discovery and Clinical Development of Antiretroviral Therapies from December 13-17, 1998. In addition to plenary talks from invited speakers, the conference will feature oral presentations based on selected abstracts and scientific poster sessions. Topics include drug design and discovery, chemistry and preclinical development, pharmacology, virology and drug resistance, and clinical development (phase I/II/III and novel combination therapies).

Registration is limited, and preference will be given to those delegates who submit an abstract. For further information, contact the International Medical Press; tel: 404-233-6446; fax 404-233-2827; e-mail: ICDCD@intmedpress.com; or Website: http//:www.intmedpress.com/ICDCD.

### Erratum

### Vol. 4, No. 2

In the article, "Accommodating Error Analysis in Comparison and Clustering of Molecular Fingerprints, by H. Salamon, M.R. Segal, A. Ponce de Leon, and P.M. Small, in Table 1 on page 162, mean kilobases for H37Rv band 12 should be 0.936.

Preventing Emerging Infectious Diseases: A Strategy for the 21st Century outlines new measures toward achieving emerging infectious disease prevention and control. The updated plan signals the second phase of the campaign launched in 1994 with the publication of Addressing Emerging Infectious Disease Threats: A Prevention Strategy for the United States, a collaborative effort of the Centers for Disease Control and Prevention under the leadership of the National Center for Infectious Diseases and institutions and agencies throughout the United States and abroad.

The objectives and activities in the updated plan are organized under the same four goals described in the 1994 publication: surveillance and response, applied research, infrastructure and training*, *and prevention and control. Nine specific priority program areas are outlined: antimicrobial resistance; foodborne and waterborne diseases; vector-borne and zoonotic diseases; diseases transmitted through blood transfusions or blood products; chronic diseases caused by infectious agents; vaccine development and use; diseases of people with impaired host defenses; diseases of pregnant women and newborns; and diseases of travelers, immigrants, and refugees.

Achieving the goals outlined in the updated plan will continue to require sustained and coordinated efforts of agencies and organizations, state and local health departments (surveillance of infectious diseases), academic centers and other federal agencies (research), health-care providers and health-care networks (guideline development and dissemination), international organizations (outbreak responses overseas), and other partners.

The executive summary of Preventing Emerging Infectious Diseases: A Strategy for the 21st Century will be released as a special issue of the Morbidity and Mortality Weekly Report on September 10, 1998. An electronic version of the full document as well as information on how to order a print copy will be available shortly afterwards at http://www.cdc.gov/ncidod/ncid.htm.

Update May 11, 2010: An electronic version of the full document is available at http://www.cdc.gov/mmwr/preview/mmwrhtml/00054779.htm. You may download a PDF version of the report at http://www.cdc.gov/MMWR/PDF/rr/rr4715.pdf. 

